# Natural Compounds as Antimicrobial Agents—2nd Edition

**DOI:** 10.3390/antibiotics14030268

**Published:** 2025-03-06

**Authors:** Carlos M. Franco, Beatriz I. Vázquez

**Affiliations:** Hygiene, Inspection and Food Control Laboratory, Analytical Chemistry, Nutrition and Bromatology Department, Faculty of Veterinary Medicine, University of Santiago de Compostela, 27002 Lugo, Spain

This monograph first covers two review papers from Prof. Mickymaray on the mechanisms and efficacy of flavonoids as antimicrobial compounds [1] against opportunistic nontuberculous mycobacteria. Mickymaray and coworkers reviewed the conventional treatments for these mycobacteria and then reviewed the newly discovered mechanisms of anti-nontuberculous mycobacteria, such as the inhibition of cell wall formation, the inhibition of biofilm formation, or even the inhibition of bacterial DNA synthesis, as well as the synergies with other antimycobacterial agents. The second review covers some antimicrobial animal substances, in this case, the secretions of toad skin (Anura, *Bufonidae*) against human pathogens [2]. These authors reported MIC values as low as 4 µg/mL against some Gram-positive bacteria, such as *E. faecalis*, or even less for the secretion of some toad species. Finally, the effectiveness of these secretions as antifungal or antiprotozoal agents is also revealed.

This research begins with a study in our laboratory with respect to the antimicrobial activity of several apitoxins. The apitoxin was directly collected from five apiaries in Ecuador, and the effects of these compounds on different *Salmonella* strains, including not only different serotypes from *Salmonella enterica* subsp. *Enterica*, but also *Salmonella enterica* subsp. arizonae and *Salmonella enterica* subsp. salamae isolates, were studied. Specifically, the relatively high number of different isolates assayed from this genus proved to be one of the most important aspects of this research; previously, the effects of Apitoxin on motility, biofilm formation and gene expression were reported [3]. There is an interesting paper by Brazilian researchers directed by Prof. Viana Pontual regarding the in vitro immune response of peripheral blood mononuclear cells infected with *Trypanosoma cruzi*, the etiological agent of Chagas disease [4], that uses a protein with activity and toxicity to *T. cruzi* from *Moringa oleifera*, a pantropical tree, that is more toxic to the parasite than to immune human cells. This issue also addresses the activity of insect compounds, specifically those of the assassin bug (*Rhynocoris iracundus*) against *Schistosoma mansoni* [5], the venom of *Rhynocoris iracundus*, which inhibits the motility of adult worms of *Schistosoma*, as well as their ability to produce eggs; thus, this venom contains potential antischistosomal compounds. In another extraordinary paper, Shcherbakova et al. studied the sensitization of fungi to 6-demethylmevinolin, a putative natural sensitizer of microbial origin that is able to suppress the biosynthesis of aflatoxin B1 [6], to avoid the frequent increase in fungicide resistance or the use of higher dosages of fungicides in the fight against the fungi cited below. By using this sensitizer, it was possible to show how the former effects colony growth as well as the conidial germination of several vegetal pathogens, such as some *Alternaria*, *Parastagonospora*, *Rhizoctonia*, and *Fusarium* species. These authors confirmed the possibility of using the sensitizer mentioned above instead of using triazole- and strobilurin-based fungicides. In addition, there is one more paper from Prof. Durant-Archibold’s group regarding the antibacterial activity of volatile organic compounds produced by the octocoral-associated *Bacillus* sp. BO53 and *Pseudomonas* GA327, thus, revealing for the first time the effects of volatile organic compounds produced by these agents associated with corals against important human pathogens, such as methicillin-resistant *S. aureus*, *Acinetobacter baumanni*, and *Pseudomonas aeruginosa*, which in turn are ESKAPE bacteria, due to their easy presentation of resistance. However, these bacteria have previously been shown to have antimicrobial properties [7]. The following paper is a very interesting one, dealing with L-carnitine effects used to grow rabbits, presenting the growth efficiency, hematological, biochemical, and carcass aspects. This work is unique from my point of view because of the nature of carnitine, which is naturally synthesized in the liver, kidneys, or brain, from lysine and methionine, and has shown interesting antimicrobial properties not known until now [8]. Zhang and coworkers studied the inhibition of *Clostridium perfringens* by *Polygonum hydropiper* compound extract, a known plant with various therapeutic properties that previously exhibited activity against other pathogens [9]. These authors also elucidated the effect on the intestinal inflammatory response by modulating NLRP3 inflammasome signaling with various flavonoids as key active compounds. The last published paper is related to the effect of fennel byproduct extracts applicable to meat products. This Italian study by Raffaele Marrone explored the use of fennel waste extracts with both antioxidant and antimicrobial properties [10] against several pathogens, such as *Salmonella*, *E. coli*, and *S. aureus*. They conclude that the cited extracts may be used to preserve the quality of beef burgers. In summary, this Special Issue contains eight research papers, two of which focus on several aspects of the fight against parasites, one of which focuses on the fight against fungi, four of which focus on the fight against bacteria, and one that focuses on the effects of carnitine on several productive parameters during rabbit growth.
